# Associations between biological maturation, physical performance, postural control, and mathematical achievement in youth soccer players

**DOI:** 10.1371/journal.pone.0298301

**Published:** 2024-03-07

**Authors:** Souhail Hermassi, Ferman Konukman, Senaid Salem Al-Marri, Lawrence D. Hayes, Thomas Bartels, René Schwesig

**Affiliations:** 1 Physical Education Department, College of Education, Qatar University, Doha, Qatar; 2 Sport and Physical Activity Research Institute, School of Health and Life Sciences, University of the West of Scotland, Glasgow, United Kingdom; 3 MVZ Sports Clinic Halle GmbH, Center of Joint Surgery, Halle (Saale), Germany; 4 Department of Orthopedic and Trauma Surgery, Martin-Luther-University Halle-Wittenberg, Halle (Saale), Germany; Instituto Politecnico de Setubal, PORTUGAL

## Abstract

This investigation explored relationships between biological maturation, physical and academic performance in young male soccer players. Thirty-eight players (age: 9.79 ± 1.21 years; body mass index (BMI): 20.4 ± 2.39 kg/m^2^; body fat: 16.8 ± 2.21%) participated. Measures of anthropometry used for body mass, body fat percentage (%BF), and BMI as well. Postural control, 15 m sprint, squat jumps and counter-movement jumps (SJ, CMJ), and T-half test for change-of-direction (CoD) were parameters of physical performance. The grade point average (GPA) of mathematics determined academic attainment. Moore’s equations were used to estimate their maturity status (PHV). Biological maturation was highly correlated with most (not 15 m sprint) physical and academic performance parameters, especially CMJ (r = -0.812) and mathematics (r = -0.781). Academic performance showed the largest relations to the jumping performance (CMJ: r = 0.771; SJ: r = 0.723). In contrast, anthropometric and fatness parameters were not relevantly (r ≥ 0.5) correlated with any other parameters. The largest correlations were calculated for sitting height vs. SJ (r = -0.408), sitting height vs. postural control (r = -0.355), leg length vs. postural control (r = -0.339). As a result, it is essential to take biological maturation inconsideration while assessing the physical and academic achievement of young soccer players. In consequence, soccer coaches and physical education (PE) teachers should be cognizant of the impact of biological maturity on physical and academic performance to assist fair and equal opportunities for achievement in young players.

## Introduction

In youths, sport participation has a number of both negative and positive effects on young team sport players [1–4]. Benefits include superior mental health, overall health, improved self-confidence amongst others [2–6]. Conversely, a negative aspect is the association of practicing sports activity with the contingency of injuries [4, 7, 8]. Numerous young team sport athletes resign from a sport at the age of 15 years of age [1, 3]. Team soccer success is reliant upon numerous performance characteristics, either soccer-specific or transferable, tactical game, physiological, mental and cognitive [3–6]. In youth sport, players require development in various physical domains to reach elite status [6]. Besides, there is research evidence to suggest that successful physical soccer performance inquires information processing to compete in dynamic environments and with complicated mobility [6, 8], emphasizing the significance of cognitive skills in young soccer players. In this context (e.g., valid data interpretation), it is important to consider the current status of biological maturation of young athletes.

The process of biological maturation affects all tissues, organs, and systems of the body. To track the development of related to processes of maturity (the mature state), results are monitored and/or measured [9]. The level of improvement at the observation’s chronological age (CA) is used to measure maturity, and the timing CA related to maturational activities take place. Although they are related, they are not analogous [10, 11]. Rate of maturation is a related aspect but is difficult to estimate. For example, muscle power and strength development promptly increase during the progress of maturity until the age of 20 years of age [5]. Consequently, growth and maturation levels are crucial in terms of evaluating youth soccer players’ physical and technical competencies [6, 8]. Especially sensomotoric (e.g., postural control and regulation) and speed aspects play an important role in this phase of biological maturation.

Postural control is important for changing direction performance [12–14]. It has been reported that soccer players with high quality postural stability develop different postural stability strategies [12] and rely less on vision [13]. Some soccer training programs are favorable for postural control [15, 16]. Cognitive ability, including core and higher-level cognitive functions such as decision-making are characteristics of soccer skills [1–6]. Specific studies explored separate associations either cognitive functions or motor skills with the physical success of top-level soccer players [3–6]. They compared soccer players depending on performance level (amateur vs. top level) and maturity status (adolescent vs. adult athletes).

A further important aspect especially for adolescent athletes is the relationship between physical and academic performance. In addition to performing better in their chosen sport, children who possess superior physical performance also achieve more academically [3]. Various studies showed positive association between academic and physical performance [3, 17]. Children who possess superior physical performance also achieve more academic success [3, 18, 19]. Furthermore, physical activity is known to improve attention through release of transmitters involved in cognitive processes [20]. Thus, low physical activity or physical fitness levels appear to be correlated with poorer cognitive skills [21]. Higher physical activity levels are associated with higher executive functioning performance in different conditions of the Erikson flanker task, and also with higher P300 (P3) wave amplitudes of the event-related potential (ERP) in response to stimuli. This fact suggests that a stronger allocation of attentional resources during stimulus encoding is related to better performance in more physically fit children [22]. Possible physiological mechanisms of exercise-induced neural adaptation are the promotion of cerebral vascularisation, up-regulation of genes associated with cellular plasticity, increased regional blood flow, and increased levels of brain-derived neurotrophic factor (BDNF) [22].

Therefore, school-based physical performance programs are necessary to improve physical and academic performance and consequently mental health of children of all ages. In addition to the transferrable skills of physical performance influencing academic performance, physical performance has also been associated with improved cognitive ability (probably permitting greater academic performance). This improved cognitive ability also exerts direct, positive effects on specific sporting performance, serving dual purposes [17]. Other physical performance parameters such as muscle power, sprint and agility, their relationship with academic attainment is less well researched [3] and therefore it has been proposed associations between muscular performance and cognitive health require additionally investigation [3]. A relevant association between these parameters may provide insight into the design of training programs, as well as rehabilitation. With talent development and identification in mind, it would seem pertinent to explore various cognitive processes related to school achievement in relation to biological maturation in young soccer players.

Based on the described research situation considered in youth soccer players, the present investigation explored relationships between different physical performances (e.g., postural control, sprint and jumping performance) and biological maturation in youth soccer players. It was hypothesized biological maturation would be correlated to physical performance parameters and mathematical attainment.

## Materials and methods

### Participants

Thirty-eight youth male players from a soccer academy in Doha, Qatar participated (age: 9.79 ± 1.21 years; body mass: 42.1 ± 5.3 kg; height: 1.44 ± 0.06 m; BMI: 20.4 ± 2.4 kg/m^2^; body fat: 16.8 ± 2.2%). Participants were youth-academy-level schoolchildren from a soccer academy. Participants had at least 2 years soccer playing experience. Recruitment was started on 15 November 2021 and completed 15 December 2021 in an in-season soccer period (recruitment period). They self-reported no musculoskeletal injuries in the 4–6 weeks before study commencement (inclusion criteria). Subjects were players who practiced soccer from a single urban soccer academy in the Doha community (Qatar). Subjects participated two to three times each week (around 3.8 hours per week), which involved matches centered on fundamental movement skills (e.g., running, walking, catching, throwing etc.), technique, and tactical training. 40% of the session’s time concerned practice of fundamental team soccer principles (team training/performance), while 60% of the session’s time was used to improve motor skills, to improve individual performance of each player.

### Ethical approval

This study was conducted in accordance with the Declaration of Helsinki. Additionally, this cross-sectional study received approval from the Institutional Review Board at Qatar University (QU-IRB 1610-FBA/21) on October 7, 2021. Prior to the commencement of the trial, participants and/or their guardians provided written informed consent or assent. Parents or legal guardians of participants were provided with a consent form and an information sheet detailing the study’s objectives. Participants, their guardians, and any relevant soccer coaches were briefed on the study procedures and their option to withdraw from the experiment.

### Inclusion and exclusion criteria

Participants were ineligible for participation if they met any of the following conditions: (1) a mental health disorder; (2) the use of medication (including antidepressants or drugs affecting the nervous system); and (3) absence of a fully completed informed consent form. Inclusion Criteria comprised: (1) Eligible participants were those who had obtained written informed consent from a parent or guardian. (2) They should be in good health, free from any contraindications to physical activity. (3) The age range of 9–11 years included individuals with no physical restrictions on exercise.

### Procedures and evaluations

After having received consent from the parents/guardians of children, two qualified researchers recorded and conducted field tests. Data collection took place during the training sessions adopted by the schedule of the Soccer Academy, testing was completed on an outdoor soccer field. All tests were conducted at the same time of day (5 p.m. to 7 p.m.), and temperature (23°C to 26°C) (time of training) [23]. Familiarization took place two weeks before the start of this study. Before each measurement, a 15 min warm up was performed and, at the end, a 10 min cool down. Also, participants consumed water *ad libitum* to ensure proper hydration during testing. The tests were applied by the same person in similar environmental conditions on an artificial grass soccer field where every team had their testing sessions. During the evaluation, participants maintained their normal habitual nutrition. In addition, participants were tested at least 4 hours postprandial. 15 m sprints, CMJ, SJ, postural control, and the T-half test for CoD. Participants underwent testing in the same order over the course of five days. On day one, a BMI and body fat % examination was done. The stork test of static balance took place on day two. Jump ability (CMJ and SJ) was finished on day three. The 15 m sprint test was conducted on the fourth day, while the agility T-half test was run on the fifth day. Physical performance test intrarater reliability has previously been reported [3, 17].

#### Anthropometry

A stadiometer and portable digital scales accurate to 0.1 cm and 0.1 kg each were used to measure height and body weight. BMI was calculated by dividing body mass by stature squared (kg·m^2^). Measurement of body fat assessment was completed using Harpenden calipers to the nearest 0.1 mm (Baty International, Burgess Hill, Sussex, United Kingdom). The four-site technique, together with age- and sex-specific equations, were used to estimate body fat %, as has already been documented in young athletes [3]. Using the following equation, the locations tested were the biceps, triceps, subscapular, and suprailiac:

$$\text{\% Body fat}=\left(4.95/\left(\text{Density}-4.5\right)\right)\;\cdot \;100$$

Where

$$\text{Density}=1.162–0.063\left(\text{LOG sum of 4 skinfolds}\right).$$

#### Biological maturity

The following gender-specific equations were applied [24] to predict participants’ maturity offset of peak height velocity (PHV): Boys: Maturity offset (years) = −8.128741 + (0.0070346 (age x sitting height)).

#### Physical performance

Tests were completed before training sessions. Using paired photocells (Racetime 2 SF, Microgate, Italy), 15 m sprints were performed. After three attempts with a rest period of six to eight minutes between each, the best achieved value (i.e., the fewest number of seconds) was used for statistical analysis. The Optojump photoelectronic system (Optojump Next, Microgate, Italy) was used to determine CMJ and SJ. The largest leap height from the four trials, each separated by 30 s of rest, was applied for statistical analysis. Postural control was measured using the Stork Balance Test [25]. T- Electronic timing sensors (photocells, Kit Racetime 2 SF, Microgate, Italy) were used to record the results of T-half test of the experiments. Subjects conducted two trials, with a three-minute break in between, and the best trial was chosen for statistical analysis [26].

#### Academic attainment

Academic attainment was determined from the academic year 2021–2022 through school records score (0 to 100) as endorsed in mathematics in the Qatar State [17, 27]. Students were graded through three assessments (oral exam, written examination, final exam) and their final grade calculated from these assessments using a weighting of each assessment. The reason for only including mathematics was due to our interest in courses of science-related subjects. It has been reported that academic achievement depends on subject, with fitness being particularly beneficial for subjects having stronger reliance on executive cognition, such as mathematics [28]. Recent studies show that children with higher cognitive flexibility levels show higher mathematics scores [28]. Executive function skills have been shown in many research studies to be related to mathematics achievement [28–30].

### Statistical analysis

All statistical analyses (including linear regression graphs) were performed using SPSS version 28.0 for Windows (SPSS Inc., IBM, Armonk, NY, USA). Prior to analysis, data were checked for normality (Shapiro-Wilk Test). Pearson’s product moment correlations determined relationships between normally distributed variables. For not normally distributed data, Spearman’s correlations were used. A correlation (r) of < 0.1, 0.1–0.3, 0.3–0.5, 0.5–0.7, 0.7–0.9, and > 0.9, was considered trivial, small, moderate, large, very large, and almost perfect, respectively [31]. r^2^ > 0.5 (explained variance > 50%) was defined as relevant and marked in bold in the results section. Regarding the sample size of n = 38, the critical value for the product-moment-correlation based on a two-sided t-test and a = 5% is r = 0.310 [32]. Based on the exploratory character of this study, only descriptive data (mean, standard deviation (SD), minimum, maximum, 95% confidence interval (CI)) were reported.

## Results

### Normal distribution

Six variables (age: p = 0.002; sitting height: p = 0.033; sprint 15 m: p < 0.001; SJ: p = 0.003; CMJ: p < 0.001; mathematics: p < 0.001) were not normally distributed.

### Anthropometric data

Table 1 (anthropometric) and Table 2 (physical and academic performance) contain the descriptive data of all used parameters and tests to use the results to evaluate other samples.

**Table 1:** Descriptive data for age and anthropometric parameters (n = 38). Values are given as mean ± SD, minimum-maximum, 95% confidence interval (CI).

Parameter	mean ± SD	Minimum-Maximum	95% CI
Age [year]	9.79 ± 1.21	8.00–12.0	9.39–10.2
Body height [m]	1.44 ± 0.06	1.30–1.62	1.42–1.46
Body weight [kg]	42.1 ± 5.34	30.0–56.0	40.4–43.9
BMI [kg/m^2^]	20.4 ± 2.39	15.5–25.6	19.6–21.2
Body fat [%]	16.8 ± 2.21	11.9–21.1	16.1–17.5
Leg length [cm]	72.3 ± 4.11	63.0–80.0	70.0–73.6
Sitting height [cm]	75.1 ± 4.15	68.0–83.0	73.7–76.4
Biological maturation PHV [year]	-2.96 ± 0.70	-4.05 –-1.12	-3.19 –-2.73

**Table 2:** Descriptive data for physical and academic performance parameters (n = 38). Values are given as mean ± SD, minimum-maximum, 95% confidence interval (CI).

Parameter	mean ± SD	Minimum-Maximum	95% CI
Sprint 15 m [s]	3.49 ± 0.33	3.09–4.12	3.38–3.60
T-half test [s]	7.70 ± 0.84	5.81–9.00	7.42–7.97
SJ [m]	27.0 ± 6.83	15.0–36.0	24.7–29.2
CMJ [cm]	31.8 ± 5.49	17.0–37.0	30.0–33.6
Postural control [s]	32.3 ± 10.8	9.0–52.0	28.7–35.8
mathematics	83.4 ± 10.0	60.0–95.0	80.1–86.7

### Relationships between biological maturation, physical and academic performance

**Fig 1:**
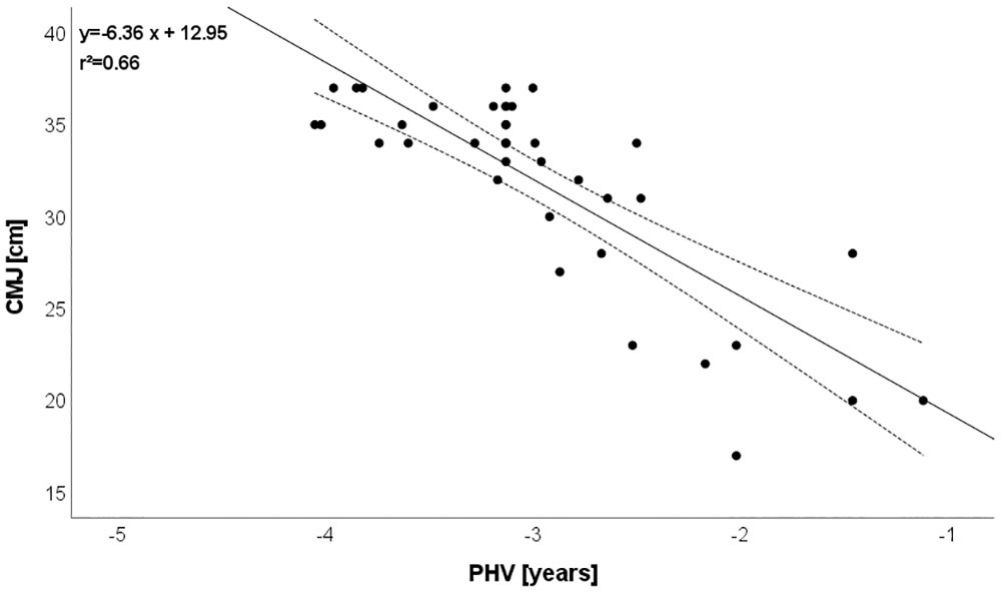
Relationship between biological maturation and CMJ. Please note that one dot can represent several subjects.

**Fig 2:**
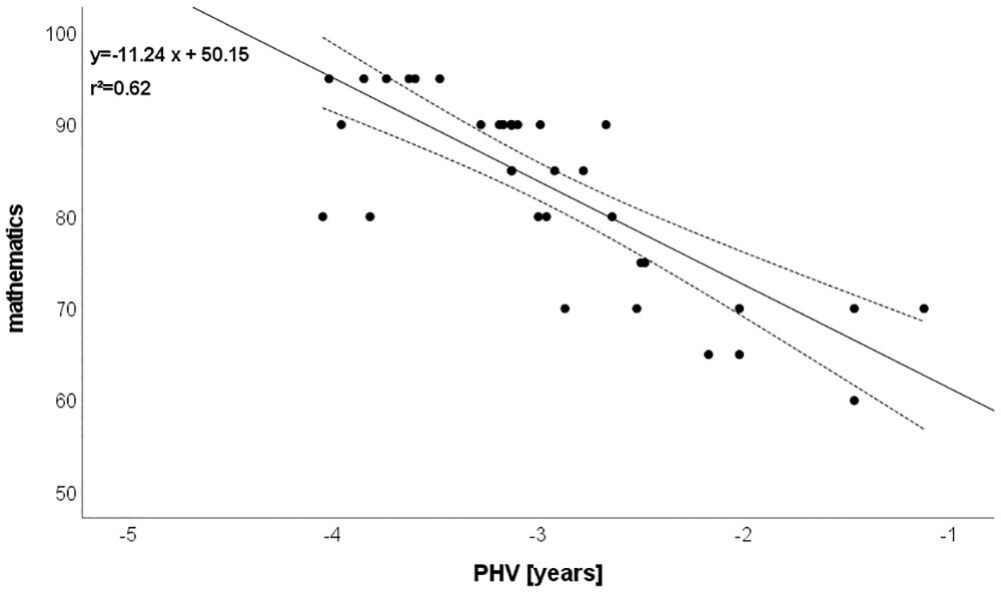
Relationship between biological maturation and mathematics. Please note that one dot can represent several subjects.

**Fig 3:**
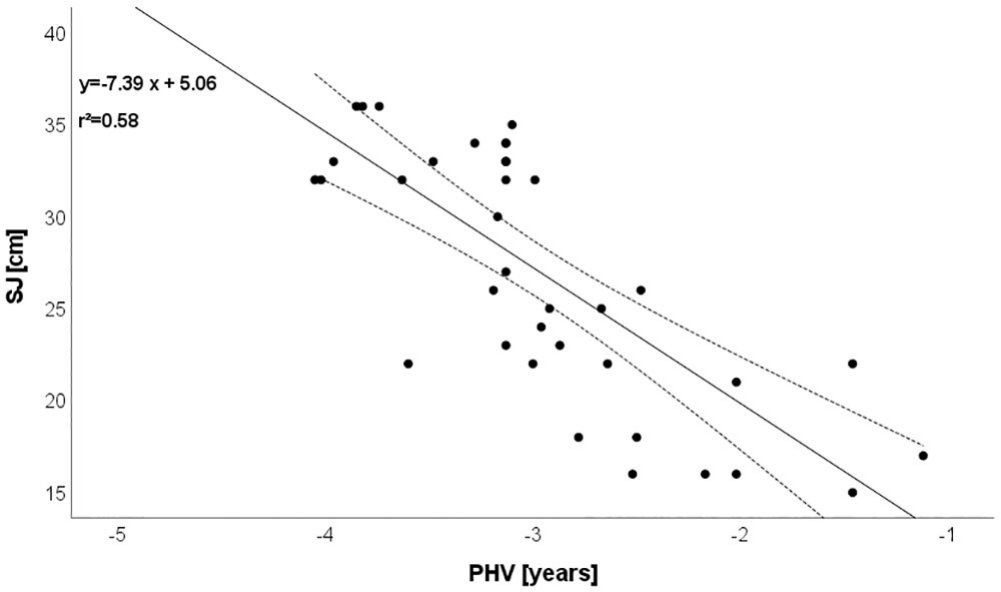
Relationship between biological maturation and SJ. Please note that one dot can represent several subjects.

**Fig 4:**
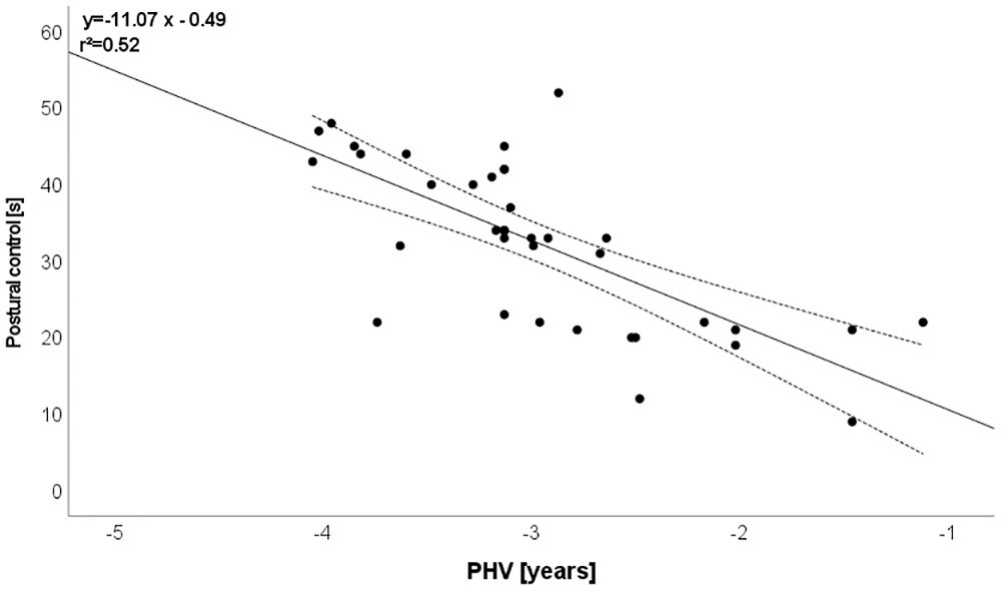
Relationship between maturation and postural control. Please note that one dot can represent several subjects.

The relationship between biological maturation (PHV) and chronological age was very high (r = 0.910). The following meaningful and relevant correlations (r ≥ 0.5) between biological maturation and parameters of different dimensions (sorted in descending order) were observed:

- CMJ: r = -0.812 (explained variance: 66%; Fig 1),
- Mathematics: r = -0.787 (explained variance: 62%; Fig 2),
- SJ: r = -0.759 (explained variance: 58%; Fig 3),
- Postural control: r = -0.719 (explained variance: 52%; Fig 4),
- T-half test: r = -0.537 (explained variance: 29%).

**Fig 5:**
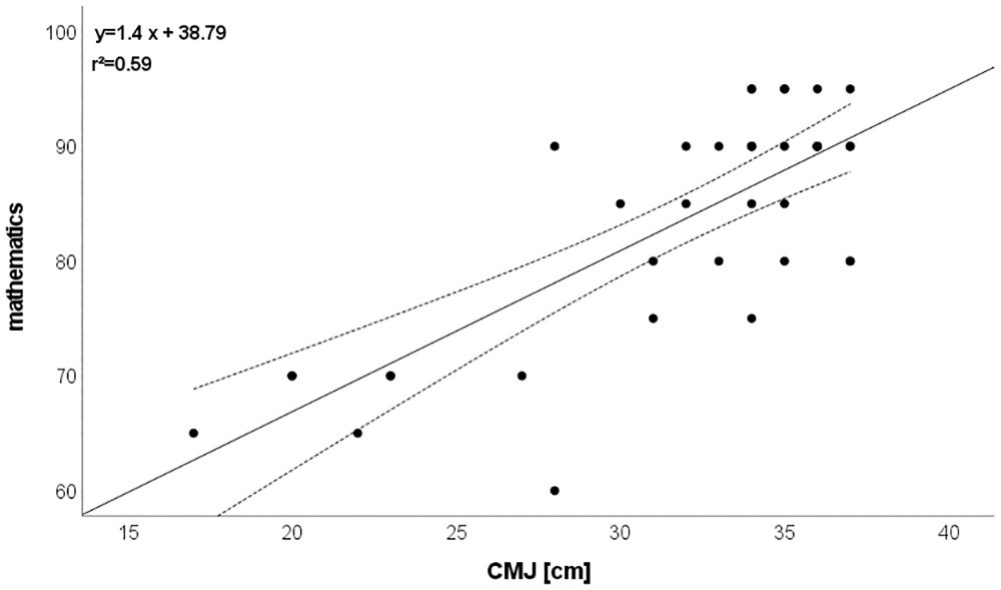
Relationship between mathematics and CMJ. Please note that one dot can represent several subjects.

**Fig 6:**
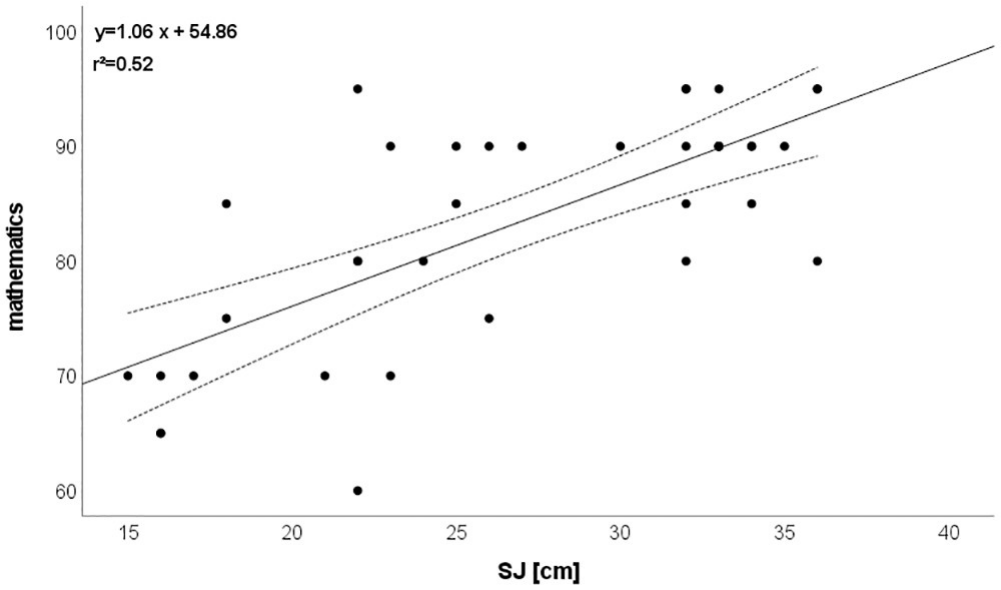
Relationship between mathematics and SJ. Please note that one dot can represent several subjects.

Academic performance (mathematics) was highly correlated with the following physical performance parameters (sorted in descending order):

- CMJ: r = 0.771 (explained variance: 59%; Fig 5),
- SJ: r = 0.723 (explained variance: 52%; Fig 6),
- Postural control: r = 0.561 (explained variance: 32%),
- T-half test: r = 0.503 (explained variance: 25%).

## Discussion

In this study, correlations between biological maturation, vertical jump, postural control, and mathematics achievement were evident in young soccer players in Qatar. Therefore, it is pertinent to note that young soccer athletes of similar chronological age can differ in biological age. This maturation difference was significantly related to a number of variables and this could therefore affect analysis of soccer skills and impact team selection [33, 34]. The main finding of this study was that physical performance, biological maturation status and mathematical achievement in youth soccer players were related with each other.

### Relationship between biological maturation and vertical jump

With age, muscle strength increases, and the adolescent growth and development periods are when the increase in muscle mass is greatest. The literature will be greatly benefited by studies on the connection between biological maturation, strength, and muscle power. This study considered the connection between biological maturation and vertical jump. For example, CMJ was highly negative correlated (r = -0.81) with biological maturation. Soccer training practice and the development of neuromuscular adaptations influence muscle strength with biological maturity. Related to researching strength and big muscles, Gillen et al. [35] indicated that variations can be linked to biological maturation [35]. However, biological maturation has been found to be highly correlated with teenage muscle strength and power [36].

Muscle power is regulated by biological maturation as a function of sex hormones that rise with the progression of puberty, according to de Almeida-Neto et al. [37], and this assertion may also help to explain the findings of the current study. It is known that in the context of neuromotor performance, biological maturation affects the maximum isometric strength (i.e., peak muscle strength) in young athletes during the puberty period [38, 39]. This is justified because, during the period of puberty, the body increases ability to recruit fast-twitch muscle fibers (i.e., fibers: II, II-a, II-b and, II-x) responsible for generating power muscular [40].

Additionally, young people with accelerated biological maturation outperformed those with delayed maturation in terms of lower limb muscle power. This is because as biological maturation advances, body mass increases, favoring the predominance of lean body mass and promoting the development of muscle strength. As a result, the data from the de Almeida-Neto et al. [37] study suggests that young athletes’ sports training should take into account the peculiarities of different biological maturation stages. Gynecomastia of biological maturation is a benign condition in males, characterized by the proliferation of glandular elements, however, hormones produced during puberty affect nearly every system within the body, causing both internal and observable changes [41, 42]. The skeletal system changes, muscles grow, the circulatory and respiratory systems undergo rapid growth and development, and nervous system changes occur. Increases in testosterone bind to androgen receptors in the limbic system, which causes a number of primary sex characteristics [41].

The results of our study are in partial agreement with some studies [43, 44]. Itoh et al. [45] reported that differences in biological maturity influenced muscle strength and lower limb power in youth soccer players. These authors concluded that soccer coaches should evaluate vertical jump performance since these characteristics were affected by maturation status of participants [46]. Nevertheless, Figueiredo et al. [46] indicated that no correlation between vertical jump and biological maturity among 11–12-year-old soccer players. The results of the current study allow us to draw the conclusion that among young soccer players, biological maturation and lower limb strength are more closely related. The results of the current study thus imply that young athletes’ sports training should take into account the peculiarities of different biological maturation phases. The fundamental causes of adolescents’ late biological maturation could be investigated in future studies.

### Relationship between biological maturation and postural control

Youth sports influence young players’ development in both positive and negative ways [47–49]. Among the advantages are increased general health, increased self-assurance, and a lower risk of mental disease. The significance of the connection between postural control and lower-extremity strength in young male top football players is underscored by this. However, postural control is fundamental for various specific soccer activities and specific technical tasks (e.g., passing and receiving, shooting, dribbling, ball control, skills and tricks, running off the ball) [50, 51]. This study indicated a relationship between biological maturity and postural control, suggesting biologically older soccer players exhibit greater postural control (explained variance: 52%, Fig 4). This is accordant with the common assumption that appropriate movement control may improve with age and experience and with practice of specific skills [52]. The weak/moderate correlations between postural control and biological maturation in our study could advocate youth soccer players demonstrate an imbalance between the level of flexibility and strength, which may lead to less coordination [53]. It has been reported that fine motor skills (e.g, control small ranges of high-precision movements like postural control) is dependent on efficient myelination [54]. Myelin greatly increases the speed of electrical communication among neurons and, hence, the brain’s computational power [55]. Therefore, myselination plays a crucial role in athletic performance. This suggests that exercise and myelin plasticity in the central nervous systems are interrelated forming a positive feedback loop [54]. These very complicated and accurate motor patterns are learned through repetition during training in young soccer players. However, the ability to improve motor skills quickly related to postural control and effectively is dependent on efficient myelination that should be developed through training process [54].

It should be added that Wilczynski et al. [56] found for youth rugby players strong positive correlations for all dynamic balance scores were associated with biological maturation. However, there were weakly positive associations between the Star Excursion Balance Test, posteromedial distance, composite distance, and maturity offset (r = 0.40, p = 0.001) as well as the latter two (r = 0.33, p = 0.004). Some studies explored the impact of biological maturation on physical performance [57], or soccer specific tasks [3]. Further studies investigated the effects of biological maturity on postural control [58, 59]. For example, Duzgun et al. [60] examined the influence the Tanner stage, an indicator of sexual maturity, exerted on one-leg-standing balance test. Holden et al. [58] explored dynamic postural control in youth players athletes in the early pubertal period by using the Star Excursion Balance Test (SEBT) and the results demonstrated no significant difference between the dynamic postural stability performance as quantified by the composite reach distance score of selected reach directions of the SEBT. However, it has been demonstrated that postural control weakly correlates to dynamic tasks in young athletes [61].

### Associations between biological maturation and mathematical achievement

It has been observed that biological maturity influences young peoples’ achievement in sport [34, 62]. According to the constraints led framework’s point of view, individual developmental differences are viewed as a constraint. These constraints interact dynamically with environmental factors like sport, school, training programs, and PE content to influence teachers’ assessment strategies [63]. In our study, associations existed between postural control and mathematics achievement. These results are in line with other studies in the same domain which have demonstrated physical performance in school is positively associated with mathematics attainment [64, 65]. Therefore, we affirm the existent scientific articles already associating executive functions to mathematics performance [28, 66]. However, as Clements et al. [66] pointed out, it is necessary to assess the degree of impact of each executive component on academic performance and specifically on the different mathematical skills.

Physical performance has been reported to influence academic achievement [67]. Past studies confirmed that exercise and regular physical activity improve specific functions and brain structures, particularly in performance of tests that rely on attentional systems and executive function [68–70]. Because of the time of instability, delay in motor coordination, or regression in the period immediately following the adolescent growth spurt [71, 72] may be a related explanation for the results of the current study.

Although most studies have demonstrated the influence of relative age on academic performance [73–75], relative age is still not a variable that is taken into account when organizing school groups or analyzing academic outcomes [76]. Urruticoechea et al. [77] reported a significant relationship between relative age effect and academic achievement in their meta-analysis. Urruticoechea et al. [77] asserted that relatively older schoolchildren in a primary school class (6–12 years) exhibitedgreater academic achievement than their relatively younger peers. Based on the existence of the relative age effect on child development, it is essential that future studies observe its prevalence and long term consequences in the educational sphere related to academic performance, taking into account these factors to determine importance of relative age in the integral development of the subject.

### Limitations

Our study was conducted in male youth soccer players. Therefore, it would be prudent to proceed cautiously when making suggestions or practical consequences for other sporting communities, especially regarding female youth soccer players. The biological maturation limits based on predictive models (e.g., measurements of body mass, stature, leg length, and sitting height) may affect the findings of this study. Moreover, the role of mental disease was not evaluated. The anticipated maturity offset and ages at PHV also increase with chronological age at prediction, have less fluctuation, and have some limitations with early and late maturing males with the original and updated equations. It should be considered as a further limitation of the study that the predictability could have some weakness considering that it was validated for others subjects and not for Qatar male youth soccer players. Similarly, this study was conducted cross-sectional, which also limits inferences around causality and temporal changes. Moreover, more studies of elite youth soccer players from other nationals are necessary to establish reference data in this context. Finally, academic attainment (mathematics) could be influenced by different teachers. Future research should make an effort to search growth changes over a protracted period of time during the development of youth soccer players.

## Conclusions

This investigation revealed that biological status might explain jump performance and to a lesser extent, postural control in the studied cohort. The present findings support the assumption that it is essential to take biological maturation into consideration when assessing soccer-specific physical performance and academic achievement of young soccer players. Based on associations presented here, it seems pragmatic to promote physical performance in this age group via physical exercise, which in turn could improve academic performance. Consequently, future studies should investigate optimizing training methods based on biological maturity to improve physical and intellectual performance of young soccer players at various stages of development. Consistent with the framework of constraints-focused theory, one promising avenue for future exploration lies in deciphering the dynamic and mutually influential inter-play among environmental factors, task demands, and individual constraints to gain a deeper understanding of the intricate relationship between students’ academic achievement, biological maturation, and physical prowess in athletes.
